# The Importance of the Pathologist’s Role in Assessment of the Quality of the Mesorectum

**DOI:** 10.1007/s11888-012-0124-7

**Published:** 2012-03-27

**Authors:** Steven L. Bosch, Iris D. Nagtegaal

**Affiliations:** Department of Pathology 824, Radboud University Nijmegen Medical Centre, PO Box 9101, 6500 HB Nijmegen, the Netherlands

**Keywords:** Rectal cancer, Mesorectum, Quality of surgery, Plane of surgery, Pathology

## Abstract

Total mesorectal excision (TME) is considered standard of care for rectal cancer treatment. Failure to remove the mesorectal fat envelope entirely may explain part of observed local and distant recurrences. Several studies suggest quality of the mesorectum after TME surgery as determined by pathological evaluation may influence prognosis. We aimed to determine the prognostic value of the plane of surgery as well as factors influencing the likelihood of a high-quality specimen by reviewing the literature. A pooled meta-analysis of relevant outcome data was performed where appropriate. A muscularis propria resection plane was found to increase the risk of local recurrence (RR 2.72 [95 % CI 1.36 to 5.44]) and overall recurrence (RR 2.00 [95 % CI 1.17 to 3.42]) compared to an (intra)mesorectal plane. Plane of surgery is an important factor in rectal cancer treatment and the documentation by pathologists is essential for the improvement of TME quality and patient outcome.

## Introduction

The development of total mesorectal excision (TME), introduced by Heald and Ryall in the early 1980s, is based on the notion that lateral mesorectal spread of small tumour foci, which are not removed in classic anterior resection, can lead to local recurrence after rectal cancer surgery [1, 2].

In a TME procedure the rectum and mesorectum are excised by precise dissection under direct vision of the avascular “holy” plane between the visceral and parietal pelvic fascia separating the mesorectal fat from the other pelvic structures [3]. Discontinuous tumor deposits and possibly involved lymph nodes present in the mesorectum are hereby removed together with the tumor.

The introduction of TME led to the reduction of local recurrence rates from 20–45 % [3], to around 10 % with TME surgery alone, and to 2.4–6 % after short-term neoadjuvant radiotherapy [4–6]. Predicting local recurrence by acknowledging the importance of lateral tumor spread led to the introduction of the circumferential resection margin (CRM). This margin, which comprises the entire non-peritonealized circumference of the resection specimen, has a relatively short, distally located anterior aspect, whereas posteriorly it has a triangular shape and runs up to the start of the sigmoid mesocolon [7]. Currently, CRM involvement is considered to be one of the key factors in rectal cancer treatment. A large number of studies, pooled in a meta-analysis by Nagtegaal and Quirke and including over 17,500 patients, showed a CRM of ≤1 mm to be a strong predictor of local recurrence (HR 2.7 [95 % CI 1.72 to 4.35]), distant recurrence (HR 2.78 [95 % CI 1.85 to 4.35]), and survival (HR 1.72 [95 % CI 1.27 to 2.27]). Moreover, after neoadjuvant therapy, CRM involvement was found to be an even stronger predictor of local recurrence (HR 6.3 [95 % CI 3.7 to 16.7]), but not distant recurrence and survival [8]. However, local and distant recurrences may also develop in patients with an uninvolved CRM.

The plane of resection created by the surgeon is another predictor of outcome that has been under investigation by pathologists for almost a decade, and which may explain part of the local recurrences in CRM-negative patients. Several authors to date have included an evaluation of the plane of surgery in their protocol. However, these studies show considerable variation in population size, study design, and results, making it difficult to appreciate the relevance of studied variables. It is the purpose of this article to critically review the current literature on the prognostic value of plane of surgery and the factors associated with achieving a satisfactory surgical specimen. A pooled meta-analysis of relevant outcome data will be performed where appropriate.

## Methods

In this review the factors influencing the plane of surgery of a resection specimen after TME for rectal cancer and the prognostic value of this plane are evaluated. A Pubmed search was performed using the keywords: “TME or total mesorectal excision” combined with “macroscopic evaluation, plane of surgery, quality of surgery or quality of mesorectum.” In addition cross referencing of relevant articles was performed. Only full text articles available in English and including an assessment of the surgical quality of the mesorectum were considered. In case of obvious overlap between studies the study with the highest number of patients was included. There was still some possible overlap of patients in some of the remaining studies, therefore the total number of patients cannot be determined exactly, however, 18 studies containing published data of between 4399 and 4469 individual patients were used. Information on outcome was given in nine of these studies (*n* = 2495).

Data was extracted and analyzed by a single investigator. For all studies in the pooled analysis the frequencies of mesorectal quality and number of events were available from the published text or tables. Relevant outcome measures are expressed as relative risks (RR) with 95 % confidence intervals, and total effect sizes are calculated using Review Manager (RevMan) (computer program). Version 5.1. Copenhagen: The Nordic Cochrane Centre, The Cochrane Collaboration, 2011.

A summary of the articles, their methodology, and primary results is given in Table 1.

**Table 1:** Studies included in the review

Study	Year	Patients (N)	Median follow-up	Study design	Neoadjuvant therapy (%)	Laparoscopic procedure (%)	Muscularis propria plane of resection (%)	Involved CRM (%)	pT4 (%)	APR (%)
Nagtegaal et al.	2002	180	25.8 month	RCT	0	0	23.9	22.7	6.1	38.8
Bretagnol et al.	2005	144	18 month	Single center prospective study	83.3 (50 Gy)	100	7	6	0	0
Breukink et al.	2005	25	N/A	Single center prospective study	100 (5x5 Gy)	100	16	12	0	0
Nagtegaal et al.	2005	205	60 month	RCT	0	0	36.1 (mesorectum)	28.7	32.4	100
							33.1 (sphincter)			
Jeyarajah et al.	2006	287	Complete 2 year	Single center prospective study	20.6 (5x5 Gy)	N/A	13.2	11.4	N/A	25.1
					6.6 (CRT)					
Maslekar et al.	2006	130	26 month	Single center prospective study	31.5 (5x5 Gy)	N/A	13	6.9	7.7	20
					22.3 (Chemo)					
Baik et al.	2008	100	N/A	Single center prospective study	0	N/A	0	12	0	21
Biondo et al.	2008	604	N/A	Multicenter prospective study with audit	61.1 (CRT)	34.6	8.1	11.6	8.8	21.5 (open)
										27.7 (lapsc)
Leite et al.	2009	127	34 month	Single center prospective study	48 (CRT)	N/A	26.8	30.7	6.6	20.5
Quirke et al.	2009	1156	3 year	RCT	48.8 (5x5 Gy)	N/A	13	11	N/A	32
Garcia-Grenaro et al.	2009	294	N/A	Single center prospective study	35.7 (CRT)	N/A	5.4	13.9	12.2	20.7
Gouvas et al.	2009	72	N/A	Single surgeon nonrandomized comparative study	43.1 (CRT)	45.8	4.2	25	11.1 (cT4)	13.9
Youssef et al.	2009	158	N/A	Single center prospective study	N/A	N/A	8.3	10.1	N/A	17.7
Baik et al.	2009	113	14.3 month	Prospective single surgeon nonrandomized comparative study	10.6 (CRT)	50.4 (lapsc)	1.8	8.0	0 (cT4)	0
						49.6 (robot)				
Chambers et al.	2009	204	N/A	Single center prospective study	54.4 (CRT)	N/A	9.8	9.8	10.3	15.7
Leonard et al.	2010	266	N/A	Multicenter audit	9 (5x5 Gy)	17.3	32	14.7	13.5 (cT4)	16.5
					65 (CRT)					
Kang et al.	2010	340	N/A	Multicenter RCT	100 (CRT)	50	5.6	3.5	1.5	12.6
Baek et al.	2010	64	20.2 month	Single center prospective study	85.9 (CRT)	100 (robot)	0	0	N/A	18.8

## Quality of Surgery: Definitions

In the CR07 trial protocol from January 1998 three grades of mesorectal surgical quality were introduced by Quirke et al. (P. Quirke, personal communication) (Table 2).

**Table 2:** Evaluating plane of surgery; mesorectum and sphincter complex (Nagtegaal 2005 [14])

Mesorectal fat envelope: possible planes of surgery
Mesorectal plane:
Intact mesorectum with only minor irregularities of a smooth mesorectal surface. No defect deeper than. No coning toward the distal margin of the specimen. Smooth circumferential resection margin on slicing
Intra-mesorectal plane:
Moderate bulk to the mesorectum, but irregularity of the mesorectal surface. Moderate coning of the specimen is allowed. At no site is the muscularis propria visible, with the exception of the insertion of the levator muscles
Muscularis propria plane:
Little bulk to the mesorectum with defects down onto the muscularis propria and/or a very irregular circumferential resection margin.
Sphincter complex: possible planes of surgery
Outside levator plane:
This plane has a cylindrical specimen with levators removed en bloc.
Sphincteric plane:
This plane has CRM on the surface of the sphincteric muscular tube, but this is intact.
Intramuscular/submucosal plane:
This plane has perforation or missing areas of muscularis propria indicating entry into the muscular tube at this level.

We [9] were the first to systematically describe the macroscopic quality of the mesorectum in rectal resection specimens from a large randomized clinical trial, and to correlate quality to outcome. We used the definitions as formulated in the CR07 protocol, but a specimen was called complete, nearly complete, or incomplete, rather than good, moderate, or poor.

**Fig. 1:**
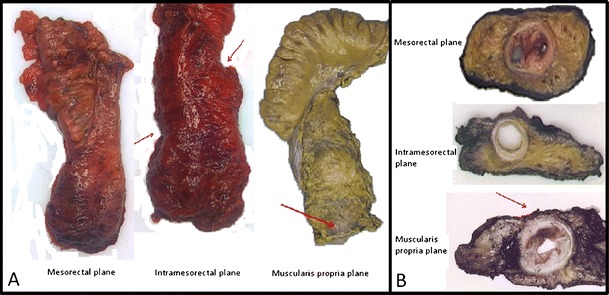
Planes of surgery. **a**, whole specimen; **b**, on slicing

In more recent publications we and others prefer an even more descriptive evaluation of mesorectal quality based on surgical plane of resection [10••, 11]. The circumferential resection margin is therefore said to be in the mesorectal plane (previously good/complete), the intra-mesorectal plane (previously moderate/nearly complete), or the muscularis propria plane (previously poor/incomplete) (Fig. 1a and b).

An underlying reason for using descriptive rather than subjective qualifications is that this method does more right to the surgeon, since there is evidence, discussed later in this review, that other factors beside surgeon competence may explain an inadequate resection plane. Furthermore, in light of increasing demands for auditing of colorectal cancer treatment it is preferable to use objective terminology that is less likely to be misinterpreted by non-medical professionals and the public.

The studies described in this review generally use the definitions as mentioned in Table 2. One study [12] uses modified definitions: an intact mesorectum is called complete, a mesorectum with injuries < 2 cm is incomplete, and a mesorectum with injuries > 2 cm is inadequate. Baik et al. [13] misquote Quirke’s definitions: “25 patients with partial injury in the fascia propria of the rectum (***less*** than 5 mm), thus of ***nearly*** complete grade.” Differences in the use of definitions may partly explain variable results between studies.

Analogous to the plane of surgery of the mesorectal fat envelope, a comment can be made on the plane of surgery around the sphincter complex after an abdominoperineal resection (APR). To date, we published the only study [14] to critically assess sphincter complex quality using the definitions in Table 2.

According to these definitions a specimen containing the levator ani muscle entirely is considered to be optimal, whereas the conventional APR specimen with the plane of resection on the sphincter complex is less than optimal, and defects in the muscularis propria of the sphincter or perforation into the lumen signify the worst grade.

As stated for the assessment of the mesorectum, the terminology for evaluating the sphincter area should be descriptive and objective.

## Incidence

Twelve studies [9, 10••, 15•, 16–19, 20•, 21–23, 24•] report frequencies of the different resection planes after open TME surgery on 3209 patients. The total percentage of mesorectal, intra-mesorectal, and muscularis propria planes was 56.4 %, 29.0 %, and 14.6 % respectively.

There is substantial variation in achieved plane of resection between studies. The five studies reporting over 70 % mesorectal plane of resection are all published after 2006. These studies are either performed in tertiary centers or specialized units [15•, 19, 23, 24•] or report results of an audited teaching program [22].

Differences between studies may be related to the wide variation in methodology regarding patient selection, interpretation of definitions, study design, and surgeon or center expertise. The time period in which the included patients were operated may influence the results because of growing awareness amongst surgeons of the importance of achieving a high quality TME. This is pointed out by Quirke et al. (2009) by reporting an improvement in plane of surgery achieved over the course of the trial [10••].

Three studies stand out as having a high percentage of intra-mesorectal and muscularis propria planes. In our study on low rectal cancer we reported the surgical quality of APR specimens only, and this may explain the high percentage of muscularis propria resection planes [14]. The results reported by Leite et al. (2009) [18] may be explained as a reflection of the individual performance of a single center, whereas Leonard et al. (2010) [20•] describe an audit of the performance of 33 potential expert surgeons from multiple centers in Belgium. Surgeons in the latter study are candidate-TME-trainers, who agreed to an external audit of their consecutive TME cases to judge whether they could serve as an alternative to foreign TME experts in a national teaching program. The fact that these are not recognized expert TME surgeons may explain a large part of the difference in achieved plane of resection with other studies. Interestingly, this study may actually give a more realistic view of average clinical practice than reports from trials by expert surgeons.

## Surgeon Experience

Variability between surgeons and centers regarding CRM involvement rates has been demonstrated repeatedly [25, 26], and can also be expected regarding the achieved plane of surgery. In the previously mentioned national audit significant heterogeneity was demonstrated when comparing 33 surgeons [20•]. However, no difference was present in two smaller studies comparing consultants with supervised registrars [16, 17]

## Laparoscopic TME

Evidence that laparoscopic resection for rectal cancer is safe and has similar short-term and long-term oncological outcome as open surgery is accumulating [24•, 27–29]. The effects of this procedure on mesorectal grade are described in eight studies [12, 15•, 20•, 21–23, 24•, 30] including 879 patients. The percentage of mesorectal, intra-mesorectal, and muscularis propria planes was 61.8 %, 23.7 %, and 14.6 % respectively.

From the eight mentioned studies, six report mesorectal plane of resection in over 70 % of cases [12, 15•, 22, 23, 24•, 30].

These studies are performed by experienced laparoscopy surgeons from specialized units, and include four single-center trials [12, 15•, 23, 30], one RCT [24•], and one multicenter observational study [22].

As was observed for open surgery, the study by Leonard et al. [20•] shows a high percentage of intra-mesorectal (35.7 %) and muscularis propria (48.2 %) resection planes.

A direct comparison between laparoscopic or open TME regarding achieved plane of surgery is made in 5 of the 8 articles. In three of those studies no difference was observed [21, 22, 24•]. One study found a better quality of surgery (as judged by the operating surgeon) in the laparoscopy arm [23], whereas in the national audit [20•] better results are reported for the open surgery arm.

A meta-analysis showed no significant difference in plane of surgery for laparoscopic versus open TME (RR 1.31 [95 % CI 0.93 to 1.84]).

## Robot-Assisted TME

Robot-assisted TME is an alternative for laparoscopy and the results of achieved planes have been studied in two study populations. Baek et al. [31] (*n* = 64) report 84.2 % mesorectal plane of surgery whereas Baik et al. [15•] compare laparoscopic and robot-assisted TME in 113 consecutive cases reporting mesorectal plane in 75.4 % and 92.9 % respectively (*P* = 0.033). These results need to be substantiated but seem to indicate that robot-assisted TME can produce a good-quality specimen.

## Anterior Resection Versus Abdominoperineal Resection

Depending on the location of the tumor and the skills of the surgeon an anterior resection (AR) or abdominoperineal resection (APR) is performed. APRs tend to have higher local recurrence rates and worse survival than ARs. This can partly be explained by higher rates of CRM involvement and intraoperative perforation (IOP), which are related to the removal of less tissue at the level of the tumour in an APR [32, 33].

As mentioned earlier the surgical quality of an APR can be evaluated at both the mesorectal as well as the sphincter level (Table 2). In our study on quality of surgery in APRs [14], we demonstrated a significant correlation between the surgical grades of the mesorectum and the sphincter (Pearson’s *R* = 0.144, *P* = 0.039).

Eight other studies [9, 10••, 13, 16–19, 20•] (*n* = 2540) compared mesorectal grades from AR and APR specimens after open TME. All studies except for Baik et al. [13] report significantly less mesorectal and more muscularis propria planes in APR compared to AR specimens. The combined effect analysis showed RR 2.53 (95 % CI 1.94 to 3.31) for achieving a muscularis propria plane after an APR compared to an AR. However, in a multifactorial analysis of 170 patients type of surgery was not an independent predictor of quality of surgery when compared to pathologic BMI, downstaging after chemoradiotherapy, and laparoscopic or open surgery [20•].

Tumor distance to the anal verge is an important aspect in the decision to perform an APR. Five studies (*n* = 997) described a significantly lower percentage of mesorectal [9] and a higher percentage of muscularis propria resection planes [17–19, 20•] in patients with tumors at < 5 cm from the anal verge compared to > 5 cm.

## Neoadjuvant Therapy

A number of clinical trials over the last 20 years have demonstrated the benefits of neoadjuvant therapy in rectal carcinoma [4, 6, 34, 35].

The effect of radiotherapy and chemoradiotherapy (CRT) on mesorectal quality was compared to no neoadjuvant therapy in six studies (*n* = 2260) [10••, 16–19, 20•]. None of the studies showed a significant difference in plane of surgery achieved between the two groups. However, in one study a small subgroup of patients that did not show downstaging after long course CRT, had a higher incidence of muscularis propria plane of resection compared to patients who did show downstaging (*P* = 0.0005 on multivariate analysis) [20•].

## Other Factors

Seven authors (*n* = 2440) make a remark on the influence of tumour extent and presence of lymph node metastases on quality of surgery. No significant relation was found with T-stage, N-stage, TNM-stage, or Dukes-stage [9, 10••, 16–19, 20•].

Data about the correlation of plane of surgery and gender are confusing. In three studies with 437 patients no correlation was found [9, 17, 18]. The plane was worse in male patients in one study [13] (*n* = 100) and in female patients in two studies [16, 20•] (*n* = 287 and *n* = 266). Based on MRI pelvimetry data it would be expected that good planes of surgery would be more difficult to achieve in patients with a relatively short interspinous distance or a short distance between sacral promontory and the top of the symphysis pubis (obstetric conjugate), as is the case in males [36].

One study [20•] found body mass index (BMI) to show a nonlinear association with the probability of a muscularis propria plane of resection (*P* = 0.003), indicating that both patients with a relatively high as well as those with a relatively low BMI are at risk. The authors state that on the one hand this indicates TME surgery is difficult in obese patients, and on the other hand little protective mesorectal fat increases the chance of accidental defects onto the muscularis propria. In contrast, Baik et al. [13] found no significant influence of BMI, but point out that the lower range of BMI values found in an Asian compared to a Western population may explain the lack of significance in this study.

Age did not influence mesorectal quality in any of the studies.

## Circumferential Resection Margin

Circumferential resection margin involvement is an important prognostic factor for the development of local recurrence, distant recurrence, and survival in rectal cancer patients. It has been associated with advanced TNM-stage, large tumor size, low tumor position, abdominoperineal resection, an ulcerative or stenosing growth pattern, surgeon experience, and on histological examination an infiltrating margin, poor differentiation, and vascular invasion [8].

The association of plane of surgery with CRM involvement has been investigated in nine studies (*n* = 2744) [9, 10••, 13, 16–19, 20•, 37]. All except one [19] of these show a significant association between achieving a muscularis propria plane of resection (combined with an intra-mesorectal plane in one study [37]) and CRM involvement. The percentage of positive margins after a muscularis propria plane of resection ranges from 19 % to 29 % in the reviewed articles whereas after a mesorectal plane these percentages range from 1.6 % to 14.6 %.

Three studies showed a significant difference in the percentage of muscularis propria resection planes between CRM-positive and CRM-negative patients: respectively 44 % versus 11 % (*P* < 0.001) [9], 30.3 % versus 7.9 % (*P* = 0.0001) [16], and 43.6 % versus 19.3 % (*P* = 0.006) [18].

Furthermore, 11.1–56.4 % of patients with CRM involvement were found to have a mesorectal plane of excision [9, 10••, 13, 16–19, 20•, 37, 38], indicating that a substantial part of CRM positivity can be explained by advanced tumor growth rather than suboptimal surgery.

## Prognosis

### Local Recurrence

**Fig. 2:**
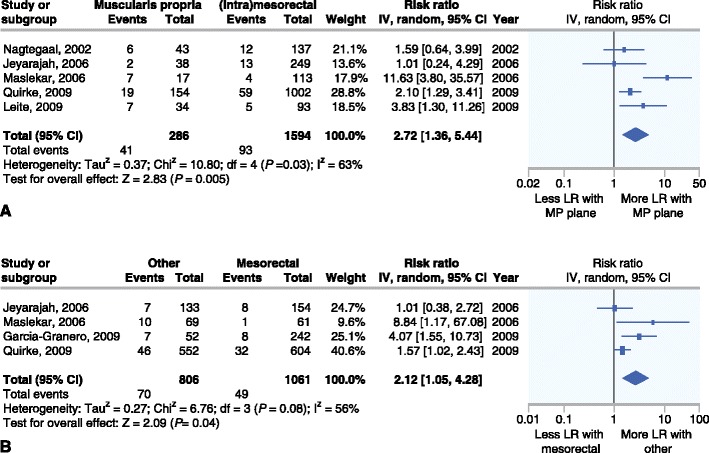
Relative risk for local recurrence after a muscularis propria versus a(n) (intra)mesorectal plane (**a**) and for local recurrence after a mesorectal plane versus both other planes (**b**)

The prognostic value of plane of surgery after open TME was described in six studies (*n* = 2174) [9, 10••, 16–19]. Four of these report a significant effect of achieved plane of surgery on local recurrence rates in a multivariate analysis [10••, 17–19]. Two studies [9, 18] combine the number of local recurrences in patients with a mesorectal and intra-mesorectal plane of resection and one study [19] combines patients with an intra-mesorectal or muscularis propria plane. Therefore, two different graphs (Fig. 2a and b) are depicted showing prognostic significance of either a mesorectal or a muscularis propria plane versus the combination of the other two planes. In the combined effect analysis patients with either a muscularis propria plane of resection have a significantly higher risk of local recurrence compared to patients with a mesorectal or intra-mesorectal plane (RR 2.72 [95 % CI 1.36 to 5.44]).

The combination of an intra-mesorectal and a muscularis propria plane of resection also significantly increases the risk of local recurrence compared to a mesorectal plane (RR 2.12 [95 % CI 1.05 to 4.28]). Furthermore, sub-analyses performed by Quirke et al. [10••] showed that patients who received neoadjuvant radiotherapy and had a mesorectal resection plane only developed local recurrence in 1 % of cases compared to 10 % of cases with a muscularis propria plane (HR 0.09 [95 % CI 0.02 to 0.49]). Moreover, CRM-negative patients showed a 4 % versus 12 % local recurrence rate for mesorectal and muscularis propria plane respectively (HR 0.33 [95 % CI 0.15 to 0.74]), indicating clinical significance of quality of surgery in this group of patients.

### Overall Recurrence

**Fig. 3:**
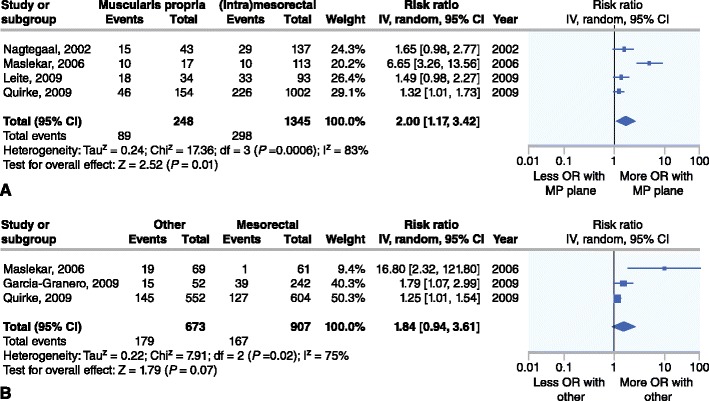
Overall recurrence after a muscularis propria plane versus both other planes (**a**) and after a mesorectal plane versus both other planes (**b**)

Five studies [9, 10••, 17–19] (*n* = 1887) report the effect of plane of resection after open TME on overall recurrence of which three show a significant difference [9, 17, 18]. In two studies [17, 18] the difference remains significant on multivariate analysis. In the meta-analysis the patients with a muscularis propria plane of resection had a significantly increased risk of overall recurrence compared to patients with a mesorectal or intra-mesorectal plane (RR 2.00 [95 % CI 1.17 to 3.42]) (Fig. 3a).

The comparison between the combined group of patients with an intra-mesorectal and a muscularis propria plane of resection and the patients with a mesorectal plane showed a trend toward significance (RR 1.84 [95 % CI 0.94 to 3.61] Z = 1.79 *P* = 0.07) (Fig. 3b).

In one study [9] CRM-negative patients were found to have overall recurrence rates of 14.9 % versus 28.6 % (*P* = 0.03) for mesorectal and intra-mesorectal versus muscularis propria plane respectively, indicating the relevance of an adequate resection plane in this subgroup as well.

### Overall Survival

Overall survival rates were only addressed in two studies (*n* = 310). In our study [9] we found survival rates of 86 % versus 76 % (*P* < 0.05) for mesorectal and intra-mesorectal planes versus a muscularis propria plane respectively, whereas Maslekar et al. [17] did not find a significant difference.

## Conclusions

We performed a meta-analysis of published data relating plane of surgery achieved after TME to patient outcome. The data consistently show that avoiding a muscularis propria plane of resection significantly reduces the risk of local recurrence and overall recurrence after TME surgery. Achieving an optimal (=mesorectal) plane of surgery also significantly improves local recurrence rates compared to a suboptimal (=intra-mesorectal or muscularis propria) plane, but for overall recurrence there is only a trend toward significance.

Worse local and overall recurrence rates after an intra-mesorectal or muscularis propria resection plane can partly be explained by CRM involvement. However, in most studies plane of surgery was a significant predictor of local recurrence in a multivariate analysis, and in CRM-negative patients it is related to local recurrence as well, indicating an independent role for plane of surgery in rectal cancer treatment.

Many factors influence the plane of resection. Heterogeneity between surgeons indicates that the skill of the surgeon is an important factor.

Type of surgery has a significant effect with APR surgery showing an inferior plane of resection more often than AR, as well as surgery on tumors at a short distance from the anal verge. In patients with either a high or low BMI it is more difficult to achieve a mesorectal resection plane.

Results from studies comparing laparoscopic to open TME suggest that laparoscopy gives at least similar quality of mesorectum as open surgery when performed by experienced surgeons, whereas less experienced surgeons may generate inferior results. Results from robot-assisted TME studies are comparable to those for laparoscopy. It seems reasonable to suggest that laparoscopic and robot-assisted TME surgery should only be performed or supervised by surgeons well beyond the learning curve. Neoadjuvant therapy does not influence achieved plane of resection.

Plane of surgery is an important factor in the treatment of rectal cancer. Pathologists have the primary responsibility to comment on resection plane in pathology reports, however, surgeons need to be aware of its importance and have to ask their pathologists for the information if it is missing. A shared responsibility for the evaluation of the mesorectum is the best way to ensure accurate feedback on surgeon performance and improvement of TME quality as well as patient outcome. Furthermore, achieved plane of surgery should be an integral part of all rectal cancer studies and audits, and should preferably be reported according to the definitions cited in this article to enable adequate comparisons.
